# Antimicrobial Polymer−Based Assemblies: A Review

**DOI:** 10.3390/ijms22115424

**Published:** 2021-05-21

**Authors:** Ana Maria Carmona-Ribeiro, Péricles Marques Araújo

**Affiliations:** Biocolloids Laboratory, Departamento de Bioquímica, Instituto de Química, Universidade de São Paulo, Av. Professor Lineu Prestes 748, São Paulo 05508-000, Brazil; periclesaraujo@usp.br

**Keywords:** cationic peptides and polymers, structure–function relationship, hydrophobic–hydrophilic balance, mechanism of cell lysis, multidrug−resistant microbes, ESKAPE pathogens, MRSA, quaternized biopolymers, antibiofilm and thromboresistant activity

## Abstract

An antimicrobial supramolecular assembly (ASA) is conspicuous in biomedical applications. Among the alternatives to overcome microbial resistance to antibiotics and drugs, ASAs, including antimicrobial peptides (AMPs) and polymers (APs), provide formulations with optimal antimicrobial activity and acceptable toxicity. AMPs and APs have been delivered by a variety of carriers such as nanoparticles, coatings, multilayers, hydrogels, liposomes, nanodisks, lyotropic lipid phases, nanostructured lipid carriers, etc. They have similar mechanisms of action involving adsorption to the cell wall, penetration across the cell membrane, and microbe lysis. APs, however, offer the advantage of cheap synthetic procedures, chemical stability, and improved adsorption (due to multipoint attachment to microbes), as compared to the expensive synthetic routes, poor yield, and subpar in vivo stability seen in AMPs. We review recent advances in polymer−based antimicrobial assemblies involving AMPs and APs.

## 1. Introduction

**Figure 1:**
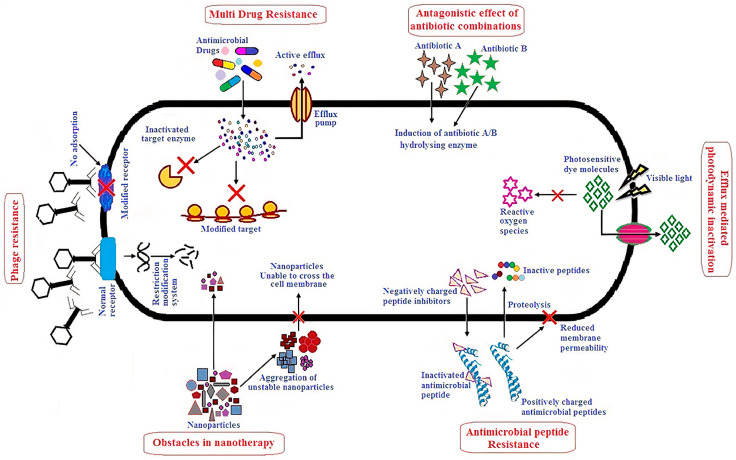
Alternative approaches to overcome multidrug−resistant (MDR) microbes and their possible shortcomings. Reprinted from [1].

Antibiotic−resistant pathogens have been considered a major menace to humans [1] so that a variety of combinatory anti−pathogenic therapies have emerged [2,3,4]. Antibiotics have been combined with bacteriophages [5], photodynamic light therapy yielding reactive oxygen species (ROS) [6], antimicrobial peptides (AMPs) [7,8,9], nanoparticles (NPs), cationic antimicrobial polymers (APs), and cationic lipids assembled as bilayer disks, vesicles, or micelles [10,11]. In general, alternative/novel therapies against multidrug−resistant (MDR) pathogens have shown promising in vitro results, but overcoming their in vivo drawbacks has remained a central challenge. Figure 1 illustrates some limitations in the way of alternative approaches.

An antimicrobial supramolecular assembly (ASA) has been opening new horizons in terms of allowing for optimal as well as broad antimicrobial activity [1,12,13,14,15,16,17,18,19,20]. Components in ASA materials can be organic, inorganic or hybrid, acting as antibacterial agents themselves and/or as carriers for timed−release of the antibacterial agent(s). ASA formulations have included coatings [21,22,23,24], functionalized surfaces [25,26], NPs [14,17,27,28,29,30,31,32], surfactant and/or lipid dispersions such as vesicles, liposomes, lipid disks [12,13,20,33], hydrogels [34,35,36], wound dressings [37], dentistry materials [38], etc.

Several ASA modes of action have been described in the literature, such as leaching of the antibacterial agent from the material [39], killing upon contact [25,40,41,42,43,44], or preventing microbial adhesion [22,45,46]. Several ASA−delivered AMPs or APs act by penetrating the cell wall, reaching bacterial cell membranes and causing their disruption [25,47].

In this review, recent developments in ASAs employing AMPs or APs were discussed in regards to their structure, activity, and applications.

## 2. ASA with AMPs

### 2.1. Structure and Antimicrobial Activity of ASA with AMPs

AMPs have been considered as amphipathic, cationic polymers with less than 50 amino acid residues, often displaying secondary structures such as α−helix [7,8,9,48]. Their main role in the innate immune system as an indispensable line of defense against pathogens in different body parts of mammals, plants, and other animals has been well documented [9]. In humans, AMPs present in oral and nasal mucosae could activate anti−inflammatory cells to sites of damaged tissue [49]. In fish, constant exposure to various types of pathogens has led to an immune system based on AMPs [50]. The cationic character determined AMPs’ interactions with the oppositely charged bacteria cell wall and penetration in the cell membrane. Destabilization of the membrane electrochemical potential allowed AMP insertion in the plasmatic membrane of the bacteria, its rupture and bacterial cell death [7,9,51,52]. Major issues against AMPs applications have been related to AMPs’ toxicity to eukaryotic cells, poor stability in vivo with eventual degradation during transportation to their target cells and organs [32,51,53,54].

**Figure 2:**
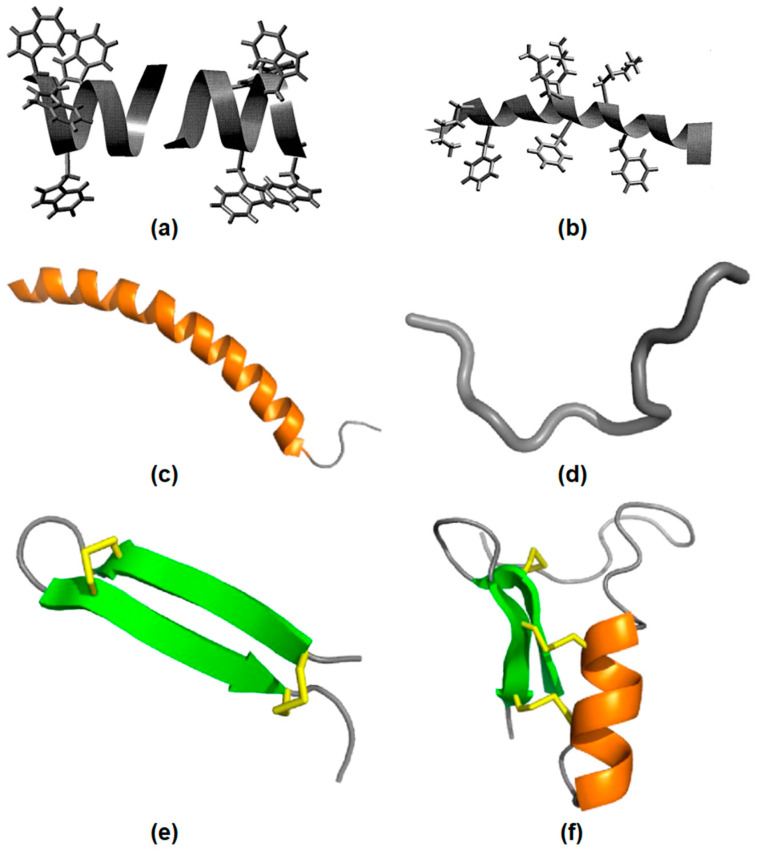
AMPs structural features. (**a**) The gramicidin A structure in membranes. (**b**) The magainin structure in micelles, adapted from [57], Copyright 1999, with permission from Elsevier. (**c**) The LL−37 peptide structure in micelles. (**d**) The indolicidin structure. (**e**) The gomesin structure stabilized by disulfide bonds. (**f**) The insect CSαβ−defensin phormicin, Adapted with permission from [58]. Copyright 2019 Elsevier.

AMPs have been classified according to their origin [8]. When they were extracted from bacteria or fungi, they belong to the nonribosomal synthetized peptides (NRAMP) class. When extracted from eukaryotic cells, they belong to the ribosomal synthetized peptides (RAMP) class. Gramicidin, vancomycin and polymyxin B are examples of NRAMPs, while nisin and melittin are RAMPs [8,55]. AMPs usually have one or more secondary structures such as α−helix, β−sheet, αβ, and non−αβ [56]. A huge structural diversity of AMPs have the common feature of positive charge and amphipathic nature [57]. Figure 2 illustrates AMPs structures. In Figure 2a, gramicidin A incorporated in bilayer membranes can be seen as a peptide dimer traversing the bilayer with four tryptophan side−chains as anchors at the membrane interface. Figure 2b shows the structure of the antimicrobial frog skin peptide magainin as determined by nuclear magnetic resonance (NMR) spectroscopy in the presence of sodium dodecyl sulfate (SDS) micelles with the side−chains of lysine and phenylalanine residues [57]. Figure 2c shows LL−37 peptide adopting a typical α−helical (orange) conformation in the presence of micelles. Figure 2d shows indolicidin in an extended conformation. Figure 2e shows the spider−derived β−hairpin peptide gomesin with β−sheets (green) typically stabilized by disulfide bonds (yellow). Figure 2f shows phormicin with both α−helix and β−sheet secondary structures [58].

**Figure 3:**
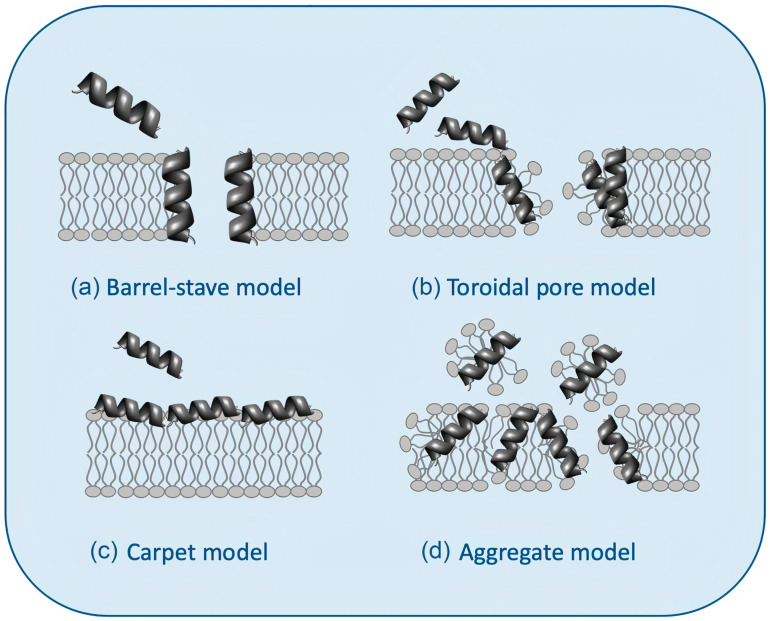
Models for the interaction between antimicrobial peptides (AMPs) and bilayer membranes: (**a**) the barrel−stave model, (**b**) the toroidal pore model, (**c**) the carpet model, and (**d**) the aggregate or “detergent−like” model was adapted from [60].

AMPs affect bacteria by inhibiting enzymatic activity, DNA or protein synthesis, or by piercing bacterial cell walls and membranes [7,9,51,52,59]. Figure 3 illustrates possible mechanisms of action for AMPs [60]. Figure 3a shows the barrel−stave model: the AMPs approach the lipid bilayer in parallel orientation but eventually penetrate it perpendicularly, keeping intermolecular peptide interactions. Figure 3b shows the “toroidal pore” model with two stages: at low concentrations (inactive state), peptides remain parallel to the plane of the bilayer; from a critical concentration, peptide molecules reorient perpendicularly penetrating the hydrophobic region of the bilayer (active state) and, together with some lipid molecules, adopt a multi−pore configuration with irreversible rupture of the plasma membrane. Figure 3c shows the carpet model: peptides remain parallel to the lipid bilayer until reaching a threshold concentration above which the membrane becomes unstable and disintegrates, forming micelles, in the so−called aggregate or “detergent−like” model (Figure 3d) [9,52,60,61,62].

The establishment of structure–function relationships for AMPs has been deemed a difficult task [63]. More than 2000 natural or synthetic AMPs with different lengths, sequences, 3−dimensional (3−D) structures and intermolecular interactions have been described. Moreover, AMPs high sensitivity to their environment has been reported from their medium−dependent−conformations. A good example is the behavior of gramicidin D (Gr) in different media [64].

**Figure 4:**
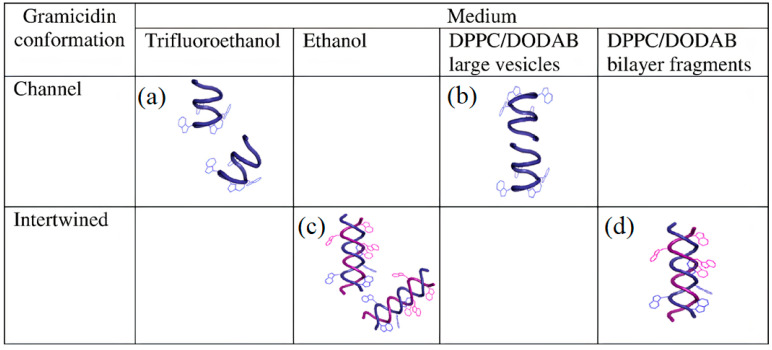
Medium−dependent gramicidin (Gr) conformation: (**a**) Gr beta−helix in trifluoroethanol, (**b**) Gr beta−helix and dimeric channel in large bilayer vesicles (LVs), (**c**) Gr intertwined beta−helices in ethanol, and (**d**) Gr intertwined beta−helices in lipid bilayer fragments (BFs). Gr molecules sense a nonpolar medium in the LV bilayer and acquire its functional channel conformation. Gr molecules sense a polar medium in the BF bilayer and become intertwined. The lipids in LVs or BFs are dipalmitoylphosphatidyl choline (DPPC) and dioctadecyl dimethyl ammonium bromide (DODAB) at a 1:1 molar ratio. Reprinted with permission from [64]. Copyright 2012 Elsevier.

Figure 4 illustrates Gr conformations in different media depicted from Gr circular dichroism (CD) and intrinsic fluorescence spectra [64]. Figure 4a,b shows Gr beta−helix in trifluoroethanol and large lipid vesicles (LV), respectively. Figure 4c,d illustrates the intertwined Gr conformation in ethanol and nanosized lipid bilayer fragments (BF), respectively. The dimeric Gr functional channel has been described as a pore spanning lipid bilayers. This pore has been associated with an ionic imbalance and bacterial cell death. Curiously, Gr channels only have been observed in LV. On the other hand, Gr intertwined dimers in the non−channel conformation only occur at the borders of cationic bilayer fragments, as shown in Figure 4d. Both LVs and BFs shield Gr tryptophans against quenching by acrylamide. However, the Stern–Volmer quenching constant is slightly higher for Gr in BFs than in LVs, confirming that the peptide was more exposed to the water medium in BFs than in LVs [64].

### 2.2. ASA with AMPs for Preserving Activity and Reducing Toxicity

An important issue regarding AMPs performance against bacteria has been the formulation [8]. The Gr behavior in different media can be used to exemplify the importance of the formulation. Furthermore, Gr formulation plays a central role not only on activity but also on toxicity, as discussed below.

Gr extracted from *Bacillus brevis* contains a group of peptides composed of 80%, 6% and 14% of gramicidin A, B, and C, respectively [64,65]. Due to Gr toxicity against eukaryotic cells, its use over a range of low concentrations has been limited to topical applications avoiding systemic administration [66,67].

In assemblies with dioctadecyldimethylammonium bromide (DODAB) bilayers, both DODAB and Gr interacted with *Escherichia coli* and *Staphylococcus aureus*. Thereby DODAB antimicrobial activity against Gram−negative bacteria [42,43] has been combined with Gr activity against Gram−positive bacteria [67]. This combination broadens the spectrum of antimicrobial activity. In addition, the toxicity against yeast eukaryotic cells of the DPDAB/Gr formulation has been tested and yielded improved yeast viability in comparison to the one of Gr alone [67].

Gr has also been formulated in lipid polymer NPs [68]. The insertion of Gr functional channels on DODAB supported bilayers has been achieved thanks to the optimization of the construction onto negatively charged polystyrene sulfate (PSS) NPs. Firstly, PSS NPs have been covered with a positively charged DODAB bilayer, which increased the zeta−average diameter by 8–10 nm, changed the zeta−potential of the NPs from negative to positive, and yielded a narrow size distribution for the PSS/DODAB/Gr NPs [68]. This formulation has been displaying broad and high antimicrobial activity at very small concentrations of the antimicrobials, namely, 0.057 and 0.0057 mM for DODAB and Gr concentrations, respectively. The results emphasized the advantages of highly organized, nanostructured, and lipid polymer cationic NPs to achieve hybrid combinations of antimicrobials with broad−spectrum activity at tiny DODAB and Gr concentrations [68]. Further applications for these Gr formulations using NPs have been envisaged in the biomedical field for treating burns, wounds, ulcers, caries, and pulp infections in dentistry, as antifouling, antimicrobial, and antibiofilm coatings on surfaces or embedded in hydrogels [8,18,35,63,66,69,70].

Since the 1960s, the AMP nisin, a RAMP lantibiotic, has been widely employed as a food preservative to extend the shelf life of dairy products (lantibiotics refers to AMPs produced by bacteria) [52,66]. In contrast to Gr, nisin has been considered nontoxic to eukaryotic cells and effective against food spoiling bacteria, showing stability over a pH and temperature range plus low susceptibility to enzymatic proteolysis [52].

Nisin has also been formulated as films released from polymer/nisin multilayers; whereas nisin/polyacrylic acid (PAA) layers disintegrated in 24 h in water solution, nisin/dextran sulfate (DX) films were stable for 14 days without releasing nisin; both films hampered the spread of *Staphylococcus epidermidis* biofilms in disk diffusion tests; therapeutic utility proposed for nisin/PAA films was treating burns and wounds due to the quick nisin release, whereas nisin/DX coatings would impart steady sterilization of surfaces over long periods of time [71].

The AMP melittin, the main component of bee venom, has been formulated on a variety of lipid or polymer based−assemblies [55,72]. In model membrane and cell culture studies, certain melittin analogues have been proposed as anticancer, antimicrobial, and low hemolytic activity [73]. The interest for this AMP has been increasing due to possible uses in a variety of cancer treatments [74,75,76,77,78,79] despite the high in vivo cytotoxicity and hemolytic activity in intravenous applications [80]. Apoptosis of cancer cells has been often reported in association with melittin; for example, cancer cell growth was inhibited via the increase of death receptor 3 expression and inactivation of NF−kappa beta in lung cancer cells [76]. A graphene formulation facilitated melittin piercing of the cell wall, causing cell lysis in Gram−negative and −positive bacteria [81].

The acronym ESKAPE pathogens have been employed to encompass E: *Enterococcus faecium*, S: *Staphylococcus aureus* or *Stenotrophomonas maltophilia*, K: *Klebsiella pneumoniae* or C: *Clostridioides difficile*, A: *Acinetobacter baumannii*, P: *Pseudomonas aeruginosa*, E: *Enterobacter spp.*, or *Enterobacteriaceae*. These MDR bacteria have been concerning physicians due to very few options left for treating infected patients; AMPs have been considered important for reversing this situation [7,82]. Recently, the synergy between antibiotics and certain AMPs has been described in a murine, sub−cutaneous abscess model caused by ESKAPE pathogens [82]. The bacteria organization on surfaces as single and multispecies biofilms has required several techniques for proper evaluation of unconventional agents, including AMPs in the treatment of biofilm infections [83,84]. Designing and optimizing AMPs will have to consider that the targets reached may not be the same; peptides could be active against several kinds of cells with activity and selectivity resulting from interaction with multiple target cell components; the cellular composition has been affecting the AMP–target cell interaction and also the design of novel AMPs [85].

Various pathogens, such as polymyxin−sensitive *Salmonella* species, have been able to penetrate macrophages, where they persisted and multiplied; modifications of NPs, liposomes, and mesoporous silica with specific cell ligands have been enabling them with penetration into macrophages and killing of intracellular pathogens [86]. Metal−based NPs, including gold NPs, have been proposed as particularly promising platforms for the intracellular delivery of AMPs, such as polymyxins eliminating intracellular *Salmonella Enterica Serovar Typhimurium* [87].

**Figure 5:**
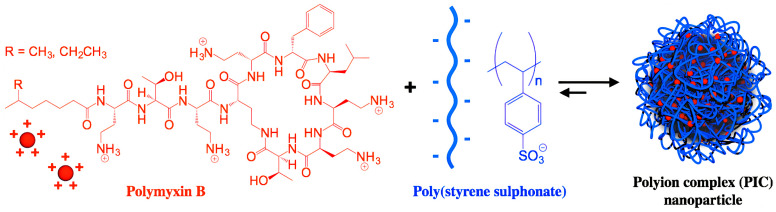
Nanoparticles of polymyxin B and poly (styrene sulphonate) active against *Pseudomonas aeruginosa*. Reprinted with permission from [90]. Copyright 2017 Elsevier.

Polymyxin has been deemed of critical medical importance against severe nosocomial multidrug−resistant Gram−negative bacteria causing nosocomial pneumonia. Several polymyxin formulations have been developed for parenteral use (for treatment of cystic fibrosis, pneumonia, bacteremia, and urinary tract infections), inhalation (cystic fibrosis, pneumonia), and topical use (optic and ophthalmic solutions). The most common polymyxin side effects have been dose−dependent nephrotoxicity and neurotoxicity; since polymyxins were essentially not absorbed by the gastrointestinal tract, their encapsulation into suitable carriers improved intestinal permeability, thereby allowing novel formulations administered by the oral route [88]. Figure 5 shows a polymer−based formulation for polymyxin B based on the electrostatic attraction between the cationic peptide and the anionic poly (styrene sulphonate) polymer as reproduced from [89,90]; the antimicrobial activity of the polymyxin AMP was influenced by the degree of polymerization of the poly−ion (DP): a low DP improved antimicrobial activity, while a high DP improved the NPs stability [89].

The relative high burden of methicillin−resistant *S. aureus* (MRSA) has been a major concern in healthcare; vancomycin, a glycopeptide antibiotic inhibiting cell wall biosynthesis, has remained the drug of choice for treatment of severe MRSA infections for many years. Unfortunately, vancomycin−resistant *S. aureus* strains have been disclosed in the 1990s; their polygenic molecular basis of resistance was due to stepwise mutations in genes encoding molecules predominantly involved in cell envelope biosynthesis [91]. Resistance has been associated with persistent infections, vancomycin treatment failure, and poor clinical outcomes. *S. aureus* strains isolated from humans, pigs, and cattle have created intermediate resistance to vancomycin [92]. Vancomycin formulated as nanoplexes of the antibiotic with dextran sulfate sodium salt has recently addressed MRSA infections; the size, polydispersity, and zeta potential of the optimized nanoplexes were 84.6 ± 4.3 nm, 0.449 ± 0.024, and −33.0 ± 4.9 mV, respectively, with 90.4 ± 0.8% complexation efficiency and 62.3 ± 0.2% drug loading; in vivo studies using a BALB/c mouse skin infection model revealed that nanoplexes reduced MRSA burden by 2.3−fold compared to bare vancomycin [93]. Liposomal vancomycin topical formulations have also produced similar results against MRSA, reconfirming the importance of the formulation for fighting drug−resistant microbia [94]. 

AMPs have been fighting not only bacteria but also other pathogens, such as fungi [95], viruses, and protozoa [15]. Besides, their versatility allowed extensions for treating from skin wounds to cancer. Therefore, future studies with AMPs are necessary, for instance, with the improvement of its stability and its scaling−up projection in the industry [51], to go beyond the combat against antimicrobial resistance. Recent advances in antimicrobial polymers in general [96] or natural and synthetic AMPs, in particular, have been reviewed [50,95,97].

Major applications for AMPs have also been the subject of important review articles such as the use of AMPs for drug design and therapeutics [98,99], natural additives for food preservation [52,100,101], prevention of caries, and pulpal infections due to dental plaques and similar others [102,103].

The most important types of carriers for AMPs were liposomes [64,67,69,70,101], nanostructured lipid carriers [104], lyotropic lipid phases (cubic and hexagonal) [105], lipid nanodisks, and bilayer fragments [10,12], NPs of several types such as biomimetic [20,28,32,68], polymeric [61,89,106], magnetic [107,108], metal−AMPs designed as metallodrugs with nuclease, and protease activity [109] or silver co−spinned with nisin in polymeric nanofibers [110], hydrogels [36,37,111], silver in alginate hydrogels [112], or fabrics [113,114,115].

**Figure 6:**
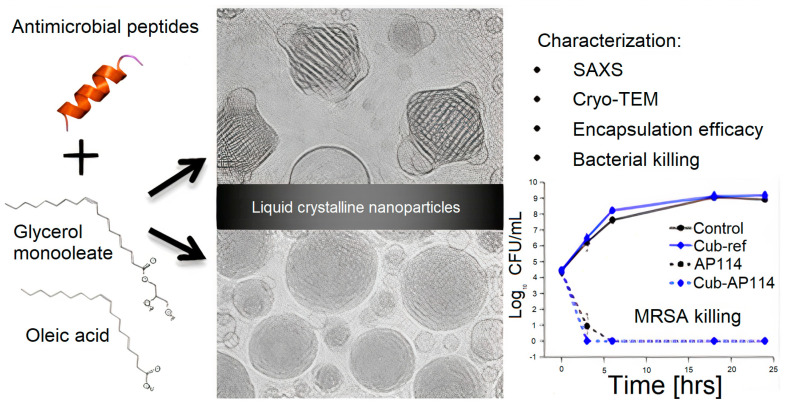
Lyotropic liquid−crystalline (LC) nanoparticles (NPs) tested as carriers for AMPs. Reproduced with permission from [105]. Copyright 2016 American Chemical Society.

Lipid−based liquid crystals as carriers for antimicrobial peptides emphasized the importance of more fluid lipid phases for the antimicrobial effect [105]. Figure 6 shows lipid−based liquid crystals from cubic and hexagonal phases; these lyotropic liquid crystalline (LC) structures consisting of cubic glycerol monooleate/water and hexagonal glycerol monooleate/oleic acid/water assemblies have been examined as carriers for AMPs. Certain AMPs had their antimicrobial activity preserved, whereas others had their activity reduced by the carriers; LC−structured gels or NPs had the capability of solubilizing both hydrophilic and hydrophobic substances, as well as being biocompatible and biodegradable; depending on AMP nature, LC showed no effect on AMPs antimicrobial activity or a diminished effect on this property.

Importantly, several AMPs formulations have been proposed against multidrug resistance. For example, liposomal AMPs combined with vancomycin exhibited improved activity against intracellular MRSA; after selecting AMPs with high antimicrobial activity, the selected peptides were lipidated, combined with model membranes (liposomes), and tested for intracellular activity against MRSA infecting human embryonic kidney epithelial cells in culture (HEK−293). They possessed good cell penetration to act against the intracellular MRSA; in addition, there was sustained release for the AMPs with a consequent improvement in the bioavailability [116].

*Mycobacterium tuberculosis* is intrinsically resistant to many antibiotics due to mutations that lead to novel strains. AMPs with metal complexes have been proposed as advantageous combinations since metal complexes associated with known AMPs often present different mechanisms of action with respect to single peptides: the destruction of bacterial plasma membranes as well as hydrolytic or oxidative cleavage of nucleic acids promoted by metal−based compounds followed from their role in the generation of reactive oxygen species able to degrade biomolecules [117]; the formulations complexing metal with AMPs could fight drug resistance against tuberculosis [118]. Adding antimicrobial and antibiofilm activities to AMPs via covalently bound metal−binding motifs improved their activities in certain cases; when combined with meropenem, streptomycin, or chloramphenicol, certain variants showed synergistic effects against *E. coli* (KpC+ 1812446) biofilms; the addition of motif also improved the survival rate of mice in a systemic infection model and reduced the hemolytic activity of the wild−type AMP [119].

Besides the AMPs, the cationic antimicrobial polymers (APs) represent another extremely promising class of antimicrobial molecules. APs are briefly presented and discussed regarding their outstanding properties in the next section.

## 3. ASAs with APs

### 3.1. Structure and Antimicrobial Activity for ASA with APs

Antimicrobial polymers (APs) have been designed to exhibit similar mechanisms of action as AMPs while diminishing AMPs’ disadvantages. APs are not degradable by enzymatic proteolysis as the AMPs, display controllable dose−dependent toxicity towards mammalian cells, have lower manufacturing costs than AMPs do, and can easily become available commercially from their facile production on a large scale following industrial synthetic protocols [48,120,121,122,123,124,125]. Most APs, similar to AMPs, are positively charged in water solution; the electrostatic attraction drives the physical adsorption onto pathogenic microbes as the first step of their mechanism of action. Thereafter, they penetrate cell walls and membranes, leading to various degrees of antimicrobial activity and toxicity that can culminate in cell lysis with leakage of internal contents [14,16,17,26]. The determination of APs specific cytotoxicity against mammalian cells has been a major requirement to establish their utility in vivo; it is important to evaluate whether APs used at a minimal bactericidal concentration (MBC) do not affect mammalian cells in culture [10,126,127]. Certain APs showed dose−dependent cytotoxicity against human epithelial cells, lung fibroblasts, and monocytes [128]. Nevertheless, their high antimicrobial activity at low doses has been the main motivation for the intense and extensive research on APs over the last twenty years [10,13,14,15,17,129]; they have been often described as a promising platform for the development of next−generation antimicrobial agents [120,122]. In addition, their flexible properties, facile synthesis, or modification from natural polymers, such as chitosan, gelatin, dextran, starch, or cellulose, also led to various alternative therapeutic applications [20,130]. Among their important applications, APs have been used as adjuvants for vaccine design and antigen presentation [126,127,129] and for gene and drug delivery [131]; interestingly, aminated microcrystalline cellulose killed melanoma and breast cancer cell lines in culture [131]. Excellent overviews on the synthesis and preparation of cationic polymers are available [48,130,132,133].

Most cationic polymers bear amine functions that can be protonated, such as polyethyleneimine (PEI), poly−L−(lysine) (PLL), chitosan, and poly [2−(N,N−dimethylamino)ethyl methacrylate] (PDMAEMA) [134]. While these polymers have inherent cationic charges, others have been developed by introducing cationic moieties such as aminated cellulose, which, compared to chitosan, represent a novel cationic cellulose derivative with improved mucoadhesive properties as well as sufficient hydration at physiological pH [135]. Chitosan derivatives bearing quaternary ammonium moieties and displaying good antimicrobial activity have also been developed [136].

**Figure 7:**
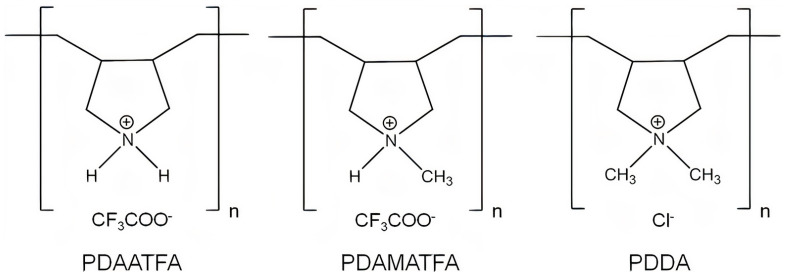
Chemical structures of some PDDA derivatives with increasing hydrophobic–hydrophilic balance from left to right. Polymers are poly (diallylammonium trifluoroacetate) (PDAATFA), poly (diallylmethylammonium trifluoroacetate) (PDAMATFA) and poly (diallyldimethylammonium chloride) PDDA. Reproduced from [17].

Antimicrobial cationic polymers mainly contain two functional components: the cationic and the hydrophobic groups. The antimicrobial activity is influenced by the type, amount, location, and distribution of these two components; the structure–function relationship for AP could provide some guidelines for developing molecular engineering of antimicrobial cationic polymers with tailor−made structures and functions [137]. For example, the chemical structures of poly (diallyldimethylammonium chloride) (PDDA) derivatives with different hydrophobic–hydrophilic balances such as poly (diallylammonium trifluoroacetate) (PDAATFA), poly (diallylmethylammonium trifluoroacetate) (PDAMATFA), and PDDA itself are shown in Figure 7 [17]; their hydrophobic–hydrophilic balance and their antimicrobial activity against Gram−negative bacteria increase from left to right [17,138].

The activities of the PDDA derivatives on Figure 7 against Gram−positive bacteria or fungus were not so clearly dependent on the hydrophobic–hydrophilic balance of the molecule, possibly due to superimposed effects of AP molecular weight and/or the nature of the molecular composition and the nature of the microbes cell wall for different species; against fungus, no effect of the molecular weight or the hydrophobic–hydrophilic balance were apparent; the fungus was very sensitive to all PDDA derivatives [16,17,138].

**Figure 8:**
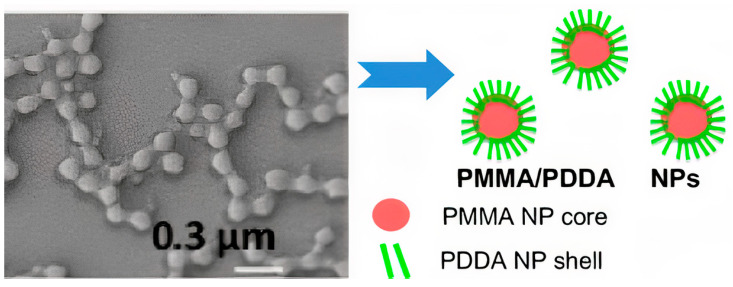
Scanning electron micrograph of poly (methyl methacrylate) PMMA/poly (diallyl dimethyl ammonium) chloride PDDA antimicrobial nanoparticles (PMMA/PDDA NPs) and schematic representation of their core−shell structure at low ionic strength. Reproduced from [17]. The NPs were obtained from emulsion polymerization of methyl methacrylate (MMA) in the presence of PDDA.

PDDA immobilization in biocompatible poly (methyl methacrylate) (PMMA) NPs diminished its antimicrobial activity to a certain extent; PMMA/PDDA NPs synthesis from emulsion polymerization of the methyl methacrylate (MMA) monomer in the presence of PDDA yielded interesting core−shell NPs; the free PDDA molecules showed lower minimal microbicidal concentrations (MMC) than the immobilized ones [17]. The core−shell nature of PMMA/PDDA NPs was an interesting finding; the hydrophobic, neutral PMMA polymeric core became surrounded by a shell of the hydrophilic, cationic polyelectrolyte PDDA, as shown in Figure 8. Apparently, the cationic NPs were not as efficient as free PDDA to penetrate the microbes’ cell walls and membranes [17,23].

**Figure 9:**
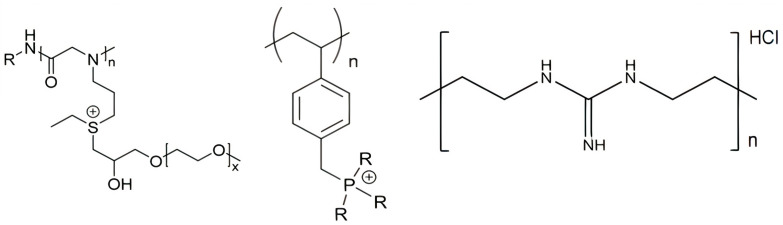
From left to right, some cationic polymers with different cationic moieties such as sulfonium [139,140], phosphonium [142], and guanidinium [141,144].

Besides the quaternary ammonium, cationic moieties in antimicrobial polymers could be sulfonium [139,140], guanidinium [125,128,141], or phosphonium [142,143]. Polymeric sulfonium salts exhibited high antibacterial activity against Gram−positive bacteria but were less active against Gram−negative bacteria [139]. It was found that the activity of the polymeric sulfonium salts was much higher than that of the corresponding monomers, particularly against *S. aureus*. Figure 9 shows some chemical structures for cationic APs with sulfonium, phosphonium, or guanidinium as the cationic moiety.

The sulfonium polypeptoid on the left (Figure 9) was obtained via ring−opening polymerization and post−modification strategy with excellent biological performance for the treatment of the infections caused by *S. aureus*; it contained both sulfonium and oligo (ethylene glycol) (OEG) motifs and displayed high selectivity for the pathogens over mammalian red blood cells [140]. Similar to natural AMPs that contain cationic and amphipathic moieties, several synthetic antimicrobial polymers named polypeptoids have been proposed. They were polymers analogous to AMPs with the advantages of facile synthesis at low cost and excellent stability against degradation in vivo. These peptidomimetic polymers had, for example, an N−substituted glycine backbone similar to the polypeptoid shown on the left in Figure 9 [140].

Various cationic polymers with quaternary ammonium or phosphonium, which possessed high antimicrobial activities in solution, exhibited a significant decrease in their antimicrobial efficiency after crosslinking or solubilization loss [17,145]. The antimicrobial activity of water−insoluble polycations could be preserved as long as the polymeric chains were long and flexible for penetration through the bacterial membranes. In a series of water−insoluble N−alkyl−N,N−dimethyl de−oxy ammonium celluloses, those modified by N,N−dimethyl dodecyl ammonium exhibited antimicrobial activity, while those modified by N,N−dimethyl butyl ammonium did not [146]. PDDA immobilization as the outer shell of PMMA nanoparticles core also reduced antimicrobial activity in comparison to the activity of free PDDA in solution [17]. Another interesting example of phosphonium−modified polymer were some inulin derivatives; inulin is a natural, renewable, biodegradable, and water−soluble carbohydrate recently modified with quaternary phosphonium salt to impart antifungal activity to the molecule. The antifungal activity increased with the alkyl chain length of the grafted quaternary phosphonium salt [147].

### 3.2. Biomedical Applications for ASA with APs

Synergistic antimicrobial activity against ESKAPE pathogens was reported for combinations of quaternary ammonium and guanidinium homopolymers [148]. Guanidinium polymers were successfully used to target intracellular, multidrug−resistant *Staphylococcus aureus* [149]. Non−leaching polyacrylate and guanidine−based copolymer NPs with 80–130 nm mean diameter were synthesized by emulsion polymerization with acrylate and glycidyl−methacrylate monomers and modified by oligoguanidine; NPs and their films presented long−term antimicrobial activity [150]. The antimicrobial copolymer of polyhexamethylene guanidine hydrochloride and polypropylene glycol diglycidyl ether adhered onto cotton fabrics both by physical adsorption and covalent binding, resulting in durable antimicrobial properties against *Escherichia coli* and *Staphylococcus aureus*; antimicrobial activity remained unchanged even after laundering the fabrics with detergent solution [151]. New chitosan derivatives bearing guanidinium functions were synthesized following different synthesis strategies. N−guanidinium chitosan acetate and N−guanidinium chitosan chloride were synthesized by direct reaction between chitosan and cyanamide in the presence of scandium (III) triflate. The synthesis of N−guanidinium chitosan (N,N′−dicyclohexyl) chloride and N−guanidinium chitosan (N−(3−dimethylaminopropyl)−N’−ethyl hydrochloride) chloride involved the reaction of chitosan with carbodiimides in ionic liquid. All newly guanylated chitosan derivatives displayed high antimicrobial activity in comparison with neat chitosan [152]. Guanidine−based polymers imparting antimicrobial activity to polysaccharides, such as cellulose, starch, and cyclodextrin, have been recently overviewed [153].

The accepted mechanism of action for cationic polymers involves the same membrane disruptive effects observed for AMPs; major events are adsorption into the bacterial cell surface, penetration into the cell wall, and insertion into the cytoplasmic membrane (due to hydrophobic group of the polymer) with membrane disruption, leakage of cytoplasmic contents, and eventually, cell lysis [14,96,121,154,155,156]. Mechanisms of action for APs and AMPs indeed decreased the odds of creating resistant bacteria [7,9,51,52,59].

Antimicrobial biopolymers were an important branch in this field; their exclusive qualities usually include being natural, biodegradable, biocompatible, cheap and extracted from biomass−derived waste, and, some of them, being both antibacterial and antifungal agents [8,157,158]. The interest in these molecules has been growing along with environmental concerns [159]. Some examples of biopolymers are: cellulose, the most abundant one in nature; chitosan, a versatile polymer attainable by treating chitin from the crustacean shell waste generated by the seafood industry; and lignin, a byproduct of the paper industry with great qualities, including antioxidant activity and high thermal stability [158,159,160,161]. Recent studies with hydroxypropyl methylcellulose (HPMC)/lignin and HPMC/lignin/chitosan films had positive results, with antimicrobial effect against both Gram−positive and Gram−negative at 35 and 0–7 °C [158]. Even though most biopolymers came with interesting advantages, some of them have their problems, such as chitosan sensitiveness to temperature and pH [162]. Biopolymers could eventually replace synthetic ones without critical side effects [159].

The biomedical applications for antimicrobial polymers required thromboresistant materials also able to avoid the formation of biofilms [163]. In biomedical devices such as catheters, intravascular grafts, extracorporeal circuits and membrane oxygenators, the adsorption of serum proteins onto these blood−contacting materials may trigger the blood coagulation cascade, whereas the contact with infective pathogens may cause the formation of biofilms and infection.

**Figure 10:**
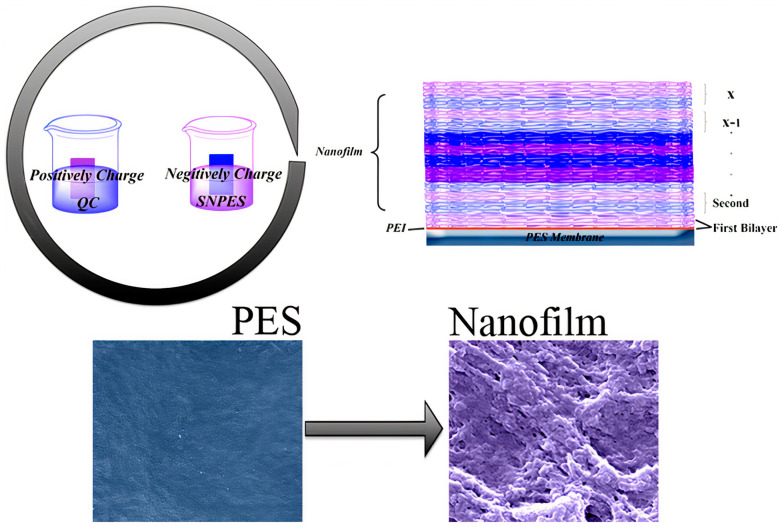
Scheme of layer−by−layer assembly of sulfonic amino poly (ether sulfone) (SNPES), a heparin−mimetic polymer, and quaternized chitosan (QC), an antimicrobial polymer, onto poly (ether sulfone) (PES) membrane substrates. SNPES and QC are negatively and positively charged, respectively. Reproduced with permission from [166]. Copyright 2015 American Chemical Society.

A few examples in the literature deal with the production of materials displaying both thromboresistant and anti−biofilm properties. The sulfonated polymers and sulfated glycosaminoglycan have been widely recognized as heparin−mimetic components since they show similar functionalities as heparin, displaying anticlotting and antithrombotic activities, the stabilization of growth factors, and the promotion of angiogenesis [164,165]. Some combined polymers joining multiple functional groups on one surface, such as a synthetic heparin−mimetic polymer or hydrophilic polymer brushes (e.g., PEG) with antibacterial quaternary compounds (QAC), were described. The layer−by−layer assembly of sulfonic amino poly (ether sulfone) (SNPES) and quaternized chitosan (QC) yielded multilayers. Additionally, when submitted to systematic tests for antithrombotic and antimicrobial activity, the multilayers showed that the heparin−mimetic multilayer−coated membrane suppressed adsorption of bovine serum fibrinogen, platelet adhesion, and activation, prolonged clotting times, and reduced activation of blood complement. Furthermore, the antibacterial test suggested that the multilayer−coated substrates exhibited activity against *Escherichia coli* and *Staphylococcus aureus* [166]. Figure 10 illustrates the preparation of these multilayered coatings.

The layer−by−layer approach has also been useful to impart anti−biofilm property to materials; deposition of PDDA and poly (acrylic acid) (PAA) layers with an outer PAA layer onto polyester fabric prevented *S. aureus* adhesion to the fabric and allowed removal of bacteria by water rinse [167].

**Figure 11:**
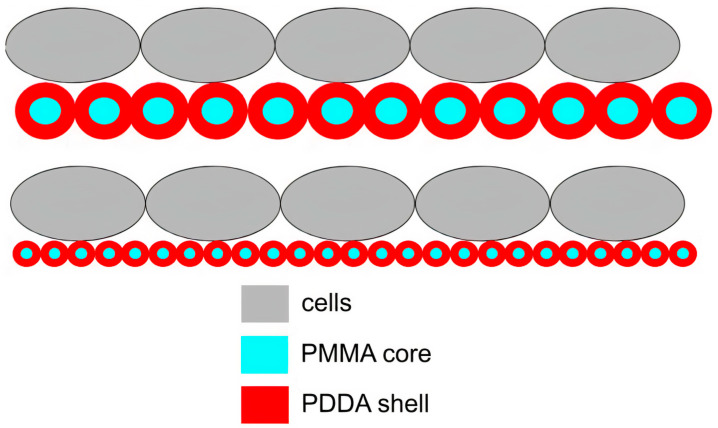
Scheme for the interaction between cells and coatings cast from core−shell nanoparticles. Nanoparticles in the coatings optimized antimicrobial activity due to a higher frequency of multipoint interactions between poly (diallyl dimethyl ammonium) chloride (PDDA) shell (in red) and cells (in grey) than the one for the larger particles. Reproduced from [31].

Recent progress in the biomedical applications of polydopamine (PDA) nanostructures, such as drug delivery, photothermal therapy, bone and tissue engineering, cell adhesion, and antimicrobial uses, were recently reviewed [168]. Rough PDA films on various substrates such as reverse osmosis filtration membranes [169], glass, plastic, stainless steel, and gauze showed remarkable antibacterial activity as compared with smooth PDA films [170]. The antifouling and antibacterial properties were attributed to the fact that the PDA film with positively charged amine groups would be responsible for the interaction with bacterial cell walls at high pH, causing cell rupture. Moreover, the rough surface of PDA exhibited higher particle contact with substrates during vigorous shaking and thus exhibited more bactericidal action [170]. Coatings cast onto silicon wafers from PMMA/PDDA nanoparticles also displayed a correlation between contact points between cells and films and the antimicrobial activity [31]. Figure 11 illustrates the compared frequency of contacts between cells and coatings from two coatings cast onto silicon wafers from PMMA/PDDA dispersions as reproduced from reference [31].

Another interesting approach recently reviewed was covalently binding or combining by physical adsorption AMPs and functional antimicrobial polymers [171], e.g., chitosan [172] or polydopamine [173]. The conjugation of AMPs into functional polymers broadened the spectrum of antimicrobial activity, including activity against MDR bacteria, reduced toxicity, and offered more functionalities for developing multifunctional biomedical hydrogels, polymer vesicles, or polymer micelles [171]. For example, the peptide anoplin, extracted from wasp venom, was covalently bound to chitosan to create a highly antimicrobial yet selective and nonhemolytic agent [172]. The conjugate displayed greater antibacterial activity when compared to anoplin only, especially against Gram–negative bacteria [172]. Another example of the peptide–polymer conjugate was a thin layer of PDA deposited onto a surface of polydimethylsiloxane (PDMS) to ease the attachment of peptide CWR11, creating a PDMS/PDA/CWR11 slide; CWR11 was attached to PDA through nucleophilic addition via thiol or amine group at either end of the peptide chain or through physical adsorption onto the PDA surface; the attachment of CWR11 conferred the PDA–coated PDMS surfaces a high antimicrobial activity against *E. coli*, *S. aureus,* and *P. aeruginosa*; the antifouling property was also present as determined by seeding fluorescently labeled–*P. aeruginosa* onto the slides of PMDS/PDA/CWR11; the material might be applied in catheters to prevent catheter–associated urinary tract infections caused by the development of biofilms on its surface [173].

**Figure 12:**
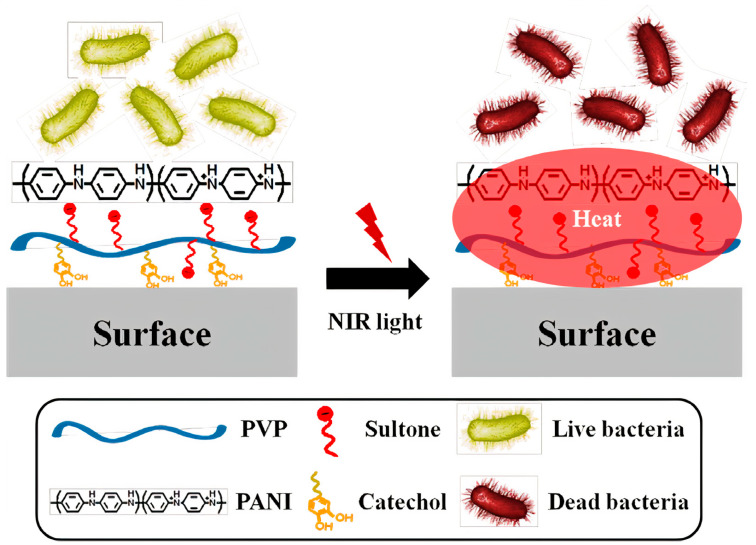
Scheme for the preparation and application of poly (vinyl pyrrolidone) sulfobetaine:poly aniline (PVPS:PANI) coating and near−infrared radiation (NIR) for the photothermolysis of bacteria. Reprinted with permission from [177]. Copyright 2015 American Chemical Society.

The conducting and hydrophilic polymer polyaniline (PANI) has been considered promising for applications in biomedicine because of its high electrical conductivity and biocompatibility. However, PANI’s low processability and degradability led to its combination with various biopolymers and nanomaterials as blends and nanocomposites, respectively. Biomedical applications of conductive PANI−based nanocomposites were available in antimicrobial therapy, drug delivery, biosensors design, nerve regeneration, and tissue engineering [174,175]. PANI materials have been used in photothermal therapy (PDT) for treating tumors or infections; the incidence of near−infrared radiation (NIR) onto PANI materials led to photothermal ablation of cancer cells or bacteria death [176]. For example, catechol−conjugated poly (vinyl pyrrolidone) sulfobetaine (PVPS) and PANI tightly linked by ionic interaction (PVPS:PANI) has been proposed as a novel photothermal antibacterial agent for surface coating, able to absorb broadband NIR light; the coating released eminent photothermal heat for the rapid killing of surface bacteria [177]. Figure 12 illustrates the photothermal effect of PVPS:PANI coatings on bacteria [177].

Similarly to PVPS: PANI coatings on Figure 12, the photothermal antimicrobial effect was also used to kill bacteria adhered to fabrics of polyethylene (PE) impregnated with poly ethylene imine−poly pyrrole NPs able to absorb the near−infrared light, thereby heating the fabric and killing adsorbed bacteria. Moreover, the fabric became washable, reusable, breathable, biocompatible, and photothermally conversable for active eradication of pathogenic bacteria [178].

A three−dimensional liver scaffold was fabricated from a chitosan/gelatin (CG) solution cross−linked with glutaraldehyde and showed a porous structure similar to the extracellular matrix that facilitated hepatocyte adhesion and proliferation; this CG scaffold had high hepatocyte biocompatibility and mechanical strength but also maintained hepatic functions and viability in in vitro cultures; especially, this liver scaffold revealed high potential for further bioartificial liver design in the near future [179].

Skin traumas such as burns and wounds are susceptible to microorganisms invasion; recent studies succeeded in treating *E. coli* and *S. aureus* infected skin with antimicrobial polymers in hydrogels, coatings, nanofibers and nanogels formulations [180,181,182,183,184]. The development of effective wound dressings was essential for speeding up wound healing.

**Figure 13:**
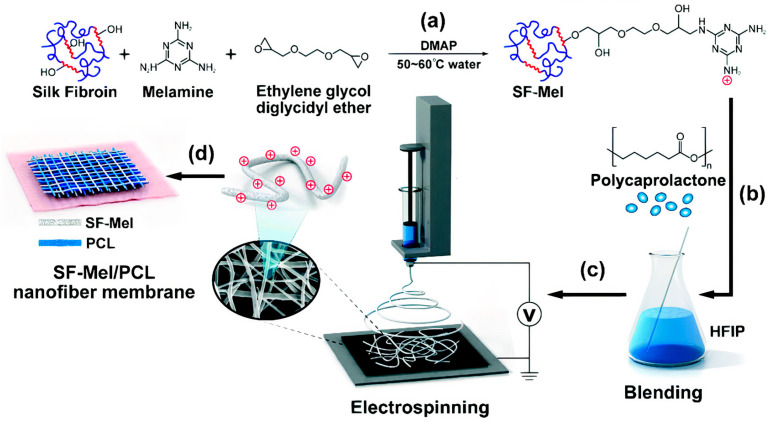
The film with cationic polymer useful as an antimicrobial wound dressing. (**a**) Melamine−modified silk fibroin (SF–Mel) was synthesized by covalent conjugation between silk fibroin and melamine. (**b**) Polycaprolactone (PCL) blended with SF–Mel enhanced the mechanical properties. (**c**) SF–Mel/PCL nanofiber films were fabricated via electrospinning. (**d**) Nanofiber membrane comprising SF–Mel/PCL was constructed as a wound dressing for skin repair. Adapted with permission from [180]. Copyright 2019 The Royal Society of Chemistry.

Rectorite, a type of layered silicate, yielded interlayered nanocomposites with positively charged polymers such as quaternized chitin; these composites combined with cellulose fibers created functional sponges with antibacterial and hemostatic properties for wound−healing applications; the in vivo animal tests demonstrated that the sponges rapidly induced hemostasis in a rat tail amputation test, making them superior to the traditional hemostatic materials; in addition, the sponges could substantially promote collagen synthesis and neovascularization, thereby accelerating wound healing 3 days earlier than gauze. This multi−functional biomedical material, fabricated using natural substances, showed great potential to be used for wound healing [185]. Another interesting antimicrobial polymer, melamine−modified silk fibroin (SF–Mel), has been used to produce films with poly caprolactone (PCL) nanofibers via the electrospinning technique. These films were hemocompatible and noncytotoxic, exhibiting broad−spectrum antibacterial activities against both Gram−negative (*Escherichia coli*) and Gram−positive bacteria (*Staphylococcus aureus*). In vivo evaluations showed accelerated wound healing by promoting re−epithelialization, collagen deposition, and vascular reconstruction; chemically grafting melamine on the side chains of silk fibroin could improve the antimicrobial properties due to the existence of positively charged amine groups derived from melamine [180]. Figure 13 illustrates the preparation of the PCL/SF–Mel wound dressings [180].

Cryopolimerization of dopamine in the presence of quaternized chitosan (QC) yielded QC/ polydopamine (PDA) cryogel, with PDA concentrations varying from 0.5 to 4.0 mg/mL so that the highest PDA concentrations yielded the best antibacterial and antioxidant activities plus near−infrared photothermal effect; moreover, these cryogels exhibited much better hemostasis than gauze and gelatin sponge in vivo in three different models: a rat liver injury model, a rabbit liver section model, and a pig skin laceration model; there was improved blood cell and platelet adhesion, with quick nonpressing surface hemostasis and wound healing [181]. Tributylammonium alginate (TBAH−Alg) salt was deposited onto modified cationic polyurethane surfaces (CPU) through supramolecular ionic interactions to create a wound dressing. Both CPU and CPU/TBAH−Alg showed large inhibition zones against bacteria in agar diffusion; in vivo experiments in wound models treated with the CPU/TBAH−Alg dressings reduced the persistent inflammatory phase and improved re−epithelialization, collagen deposition, and mature blood vessel formation, showing better results than commercial dressing Tegaderm [182].

### 3.3. Other Applications for ASA with APs

ASAs with antimicrobial polymers were also involved in the food packaging, fabrics and textile industries, in addition to water treatment [96,157].

In water treatment, the use of organic polyelectrolytes included a myriad of examples of the benefits of polymer use in conventional sedimentation and filtration; however, the influence of polymer chemical structure on performance has been investigated superficially [186]. Organic coagulants and flocculants were usually water−soluble polymers (polyelectrolytes) originated from various natural macromolecular compounds, including polyamines, PDDA, dimethylamine, and polyacrylamides [187]. Among the flocculants, only a few exhibited both antimicrobial and coagulation properties; PDDA was among those able to impart both properties to materials used for water treatment [8,13,14,17,188].

**Figure 14:**
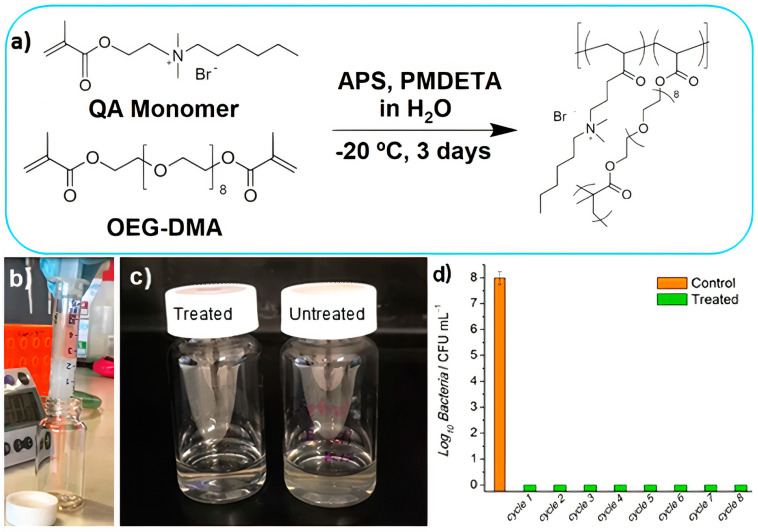
Macroporous antimicrobial polymeric gel (MAPG) for water treatment. (**a**) MAPG synthesis via free radical polymerization at subzero temperature. (**b**) Image of macroporous antimicrobial polymeric gel (MAPG) in a syringe and *E. coli*−contaminated water passing through it. (**c**) Image of treated water after passing through the syringe. Compared to the untreated, cloudy water due to the presence of bacteria, the treated water was clear. (**d**) The syringe was subjected to 8 continuous cycles of percolation with *E. coli*−contaminated water; the recovered water was analyzed via colony−forming unit (CFU) counting. No viable bacteria were detected in the water that passed through the syringe. APS is ammonium persulfate, QA monomer is the quaternary ammonium ethyl methacrylate monomer, PMDETA is the coinitiator N,N,N′,N′′,N′′−pentamethyldiethylenetriamine, and OEG−DMA is oligoethylene glycol dimethacrylate. Adapted from [189].

Macroporous antimicrobial polymeric gel (MAPG) containing quaternary ammonium in its chemical structure was synthetized through cryopolymerization; a hydrogel (HG) with the same chemical composition was also prepared for comparison. Firstly, a quaternary ammonium (QA) methacrylate monomer bearing a hydrophobic n−hexyl tail was synthesized [189]. This was an adequate antimicrobial combination of cationic and hydrophobic groups in the monomer to synthesize the polymer: the n−hexyl group was selected as sufficiently hydrophobic to cause membrane disruption, while the cationic moiety implemented adsorption to the bacteria cell wall [189]. The polymerization is shown in Figure 14. First, the QA monomer was synthesized via quaternization reaction between 2−(dimethyl amino) ethyl methacrylate and 1−bromohexane; second, the polymerization of the QA monomer via free−radical polymerization in the presence of a redox radical initiator (i.e., ammonium persulfate), coinitiator N,N,N′,N′′,N′′−pentamethyldiethylene triamine (PMDETA), and a cross−linkable monomer (i.e., oligoethylene glycol dimethacrylate (OEG−DMA)) in water was carried out at subzero temperature (Figure 14a). Filtration of contaminated water using MAPG produced pure water without bacteria (Figure 14b–d) [189]. Cryogelation required only simple mixing of chemicals, hence making the whole process potentially viable for industrial−scale preparation.

Polymers, which act as the primary substrate for face masks, could be fine−tuned to impart bio−active and bio−passive properties to the fabrics. The active moieties such as N−halamines, QACs, PEI, benzophenone (BP), polypyrrole, and inorganic groups, such as metals, have been incorporated to yield various antimicrobial polymers suitable for making a reusable facemask [190,191]. Among these, N−halamine and QACs have proven and powerful activity against a broad spectrum of microorganisms. Bath coating, spray coating, and immobilization via carriers have been employed to yield QACs modified antimicrobial fabrics. Direct polymerization of monomers and covalent attachment of QACs was expected to enhance the stability of the coating and the performance. N−halamines had high effectiveness in short contact times in antimicrobial fabrics [192]. However, the real field applicability of N−halamine on face masks has not been explored yet. Natural compounds and antimicrobial peptides are promising molecules due to less ecotoxicity and proven antimicrobial properties. Recently, the inactivation by oxygen singlet of severe acute respiratory syndrome coronavirus 2 (SARS−CoV−2) using light on synthetic conjugated polymers and oligomers was reported; five representative conjugated oligomers and polymers from an array of phenylene ethynylene−based cationic and anionic conjugated materials against SARS−CoV−2 revealed that light activation of the materials at the wavelengths where they absorb gave rise to moderate to very strong inactivation of the virus. Furthermore, no dark inactivation of the virus for three of the five materials/compounds occurred for the quaternary ammonium derivatives. Therefore, the generation of reactive oxygen species definitely inactivated the virus; the incorporation of these materials in wipes, sprays, masks, and clothing and other personal protection equipment would possibly be useful in preventing infections and the spreading of the virus and future outbreaks from similar viruses [193]. A more recent report on the development of a non−woven face mask filter fabricated with a coating of benzalkonium chloride, a quaternary ammonium compound, was able to inactivate more than 99% of SARS−CoV−2 particles in one minute of contact and also methicillin−resistant *Staphylococcus aureus* and *Staphylococcus epidermidis*; this would solve the pressing problem of commercial face masks that contained filters not capable of inactivating either SARS−CoV−2 or multidrug−resistant bacteria [194].

## 4. Conclusions

Antimicrobial polymers, such as APs and AMPs, have been widely explored as materials for biomedical applications. Their chemical structure usually contains both cationic and hydrophobic moieties, exhibiting unlimited potential to fight microbial resistance against available antibiotics. In terms of their potential shortcomings, in vivo AMPs necessitate protection from proteolytic enzymes and rapid degradation, whereas APs still require improvements in terms of their biocompatibility. The similar mechanisms of action found in APs and AMPs involve adsorption to the cell wall, penetration across the cell membrane, and microbe lysis. The synthetic procedures, chemical stability, and improved adsorption of APs—the latter due to their multipoint attachment to microbes—represent significant advantages in comparison to the expensive synthetic pathways for procedure scaling, poor yield, and subpar in vivo stability of AMPs. ASAs with APs and AMPs have also been found useful in water treatment and the production of fabrics and textiles endowed with suitable antimicrobial properties, e.g., face masks and air filters, which have become important and oftentimes crucial defenses in an era of pandemics.

## Abbreviations

NP	Nanoparticle
MDR	Multidrug−resistant
ROS	Reactive oxygen species
AMP	Antimicrobial peptide
ASA	Antimicrobial supramolecular assembly
PEG	Polyethylene glycol
NRAMP	Nonribosomal synthetized peptide
RAMP	Ribosomal synthetized peptide
DNA	Deoxyribonucleic acid
Gr	Gramicidin D
LV	Large lipid vesicles
BF	Nanosized lipidic bilayer fragments
CD	Circular dichroism
DPPC	Dipalmitoylphosphatidylcholine
DODAB	Dioctadecyldimethylammonium bromide
PSS	Polystyrene sulfate
DX	Dextran sulfate
MRSA	Methicillin−resistant *Staphylococcus aureus*
IDSA	Infectious Diseases Society of America
LC	Liquid crystalline
AP	Antimicrobial polymer
MBC	Minimum bactericidal concentration
PEI	Polyethyleneimine
PLL	Poly−L−lysine
PDMAEMA	Poly [2−(N,N−dimethylamino)ethyl methacrylate]
PDDA	Poly (diallyldimethylammonium chloride)
PDAATFA	Poly (diallylammonium trifluoroacetate)
PDAMATFA	Poly (diallylmethylammonium trifluoroacetate)
PMMA	Poly (methyl methacrylate)
MMA	Methyl metacrylate
OEG	Oligo (ethylene glycol)
HPMC	Hydroxypropylmethylcellulose
QAC	Antibacterial quaternary compound
SNPES	Sulfonic amino poly (ether sulfone)
QC	Quaternized chitosan
PAA	Poly (acrylic acid)
PDA	Polydopamine
PDMS	Polydimethylsiloxane
PANI	Polyaniline
PDT	Photothermal therapy
PVPS	Poly (vinylpyrrolidone) sulfobetaine
PE	Polyethylene
CG	Chitosan/gelatin
SF−Mel	Melamine−modified silk fibroin
PCL	Polycaprolactone
TBAH−Alg	Tributylammonium alginate
CPU	Polyurethane surfaces
MAPG	Macroporous antimicrobial polymeric gel
HG	Hydrogel
QA	Quaternary ammonium
PMDETA	N,N,N′,N″,N″−pentamethyldiethylenetriamine
OEG−DMA	Oligoethylene glycol dimethacrylate
BP	Benzophenone
SARS−CoV−2	Severe acute respiratory syndrome coronavirus 2
